# Supplementary material for: Phytocannabinoids: Chromatographic Screening of Cannabinoids and Loading into Lipid Nanoparticles
Source: Molecules. 2023 Mar 22;28(6):2875. doi: 10.3390/molecules28062875 (PMC10058297; doi:10.3390/molecules28062875)
Supplement: Supplementary file 1 [file molecules-28-02875-s001.zip › molecules-2256577-supplementary.pdf]

molecules

Supplementary Materials

Phytocannabinoids: Chromatographic Screening of Cannabinoids and Loading into Lipid Nanoparticles

Aleksandra Zielińska [1,2,*], Raquel da Ana [2], Joel Fonseca [2], Milena Szalata [3], Karolina Wielgus [4], Faezeh Fathi [5], M. Beatriz P. P. Oliveira [5], Rafał Staszewski [6], Jacek Karczewski [7,8] and Eliana B. Souto [2,9,10,*]

[1] Institute of Human Genetics, Polish Academy of Sciences, Strzeszyńska 32, 60-479 Poznań, Poland
[2] Department of Pharmaceutical Technology, Faculty of Pharmacy, University of Porto, Rua de Jorge Viterbo Ferreira, 228, 4050-313 Porto, Portugal
[3] Department of Biotechnology, Institute of Natural Fibres and Medicinal Plants, National Research Institute, Wojska Polskiego 71B, 60-630 Poznań, Poland
[4] Department of Pediatric Gastroenterology and Metabolic Diseases, Poznan University of Medical Sciences, Szpitalna 27/33, 60-572 Poznan, Poland
[5] REQUIMTE/LAQV, Department of Chemical Sciences, Faculty of Pharmacy, University of Porto, Rua Jorge Viterbo Ferreira no. 280, 4050-313 Porto, Portugal
[6] Department of Hypertension Angiology and Internal Medicine, Poznan University of Medical Sciences, 61-701 Poznań, Poland
[7] Department of Environmental Medicine, Poznan University of Medical Sciences, 61-701 Poznań, Poland
[8] Department of Gastroenterology, Dietetics and Internal Diseases, H. Swiecicki University Hospital, Poznan University of Medical Sciences, 60-355 Poznan, Poland
[9] REQUIMTE/UCIBIO, Faculty of Pharmacy, University of Porto, Rua de Jorge Viterbo Ferreira, 228, 4050-313 Porto, Portugal
[10] Associate Laboratory i4HB—Institute for Health and Bioeconomy, Faculty of Pharmacy, University of Porto, 4050-313 Porto, Portugal
* Correspondence: aleksandra.zielinska@igcz.poznan.pl (A.Z.); ebsouto@ff.up.pt (E.B.S.)

Sample 1

Chromatogram and Results

Injection Details

Injection Name:	**CT319 IGZC PAN 26.08.2020 pr. 1**	*Run Time (min)*	**22,00**
Vial Number:	**RA4**	*Injection Volum*	**2,00**
Injection Type:	**Unknown**	*Channel:*	**UV_VIS_1**
Analityk	**Dominik Duczmal**	*Wavelength:*	**230**
Instrument Method:	**metoda kanna**	*Bandwidth:*	**4**
Processing Method:	**Suma wszystkich krzywych**	*Dilution Factor:*	**100,0000**
Injection Date/Time:	**27.sie.20 00:47**	*Sample Weight:*	**131,9000**

Chromatogram

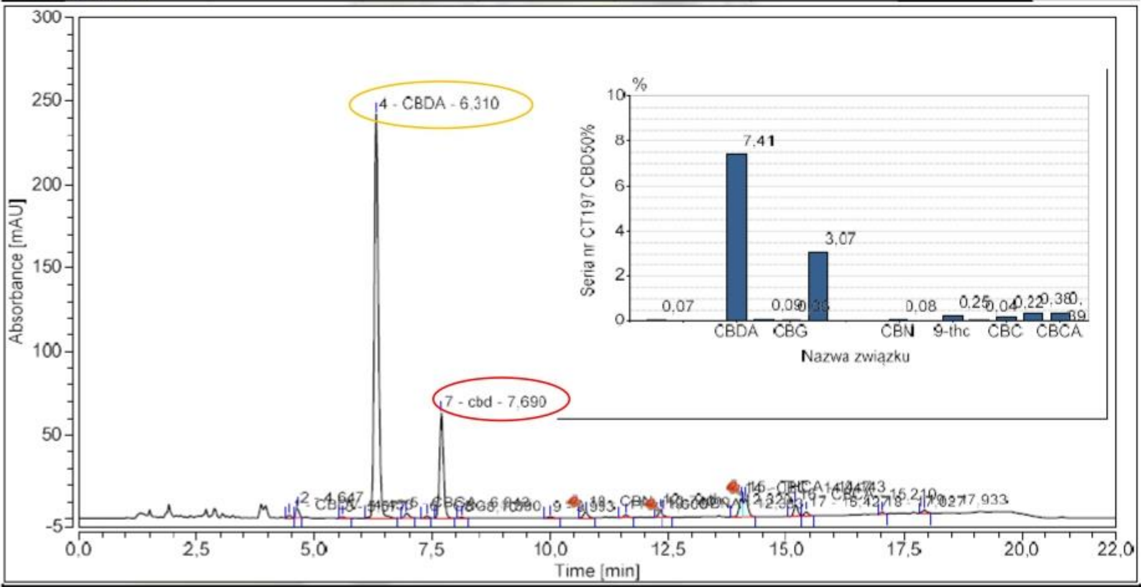

Integration Results

No.	Peak Name	Retention Time	Area	Height	Dilution	Weight	Amount	Stezenie procentowe
		min	mAU*min	mAU		mg	ug	%
1	CBDV	4,453	0,127	1,777	100,0	131,9	0,6512	0,07
2		4,647	0,467	5,979	100,0	131,9	n.a.	n.a.
3		5,577	0,093	1,059	100,0	131,9	n.a.	n.a.
4	CBDA	6,310	24,199	242,205	100,0	131,9	74,0865	7,41
5	CBGA	6,943	0,286	2,807	100,0	131,9	0,9023	0,09
6	CBG	7,390	0,104	1,137	100,0	131,9	0,5577	0,06
7	cbd	7,690	5,998	63,254	100,0	131,9	30,7084	3,07
8		8,107	0,041	0,439	100,0	131,9	n.a.	n.a.
9		9,993	0,077	0,667	100,0	131,9	n.a.	n.a.
10	CBN	10,740	0,336	3,519	100,0	131,9	0,8150	0,08
11		11,600	0,162	1,738	100,0	131,9	n.a.	n.a.
12	9-thc	12,320	0,417	4,762	100,0	131,9	2,5416	0,25
13	CBNA	12,383	0,097	1,645	100,0	131,9	0,4205	0,04
14	CBC	14,047	0,949	11,543	100,0	131,9	2,1907	0,22
15	THCA	14,143	1,177	11,680	100,0	131,9	3,8197	0,38
16	CBCA	15,210	0,637	7,083	100,0	131,9	3,8996	0,39
17		15,427	0,228	2,482	100,0	131,9	n.a.	n.a.
18		17,027	0,086	1,285	100,0	131,9	n.a.	n.a.
19		17,933	0,178	1,863	100,0	131,9	n.a.	n.a.

Figure S1. Sample 1 and chromatographic analysis of a cannabinoid extract containing a mixture of cannabinoids.

Sample 2

Chromatogram and Results

Injection Details

Injection Name:	CT320 IGZC PAN 26.08.2020 pr. 2	*Run Time (min)*	22,00
Vial Number:	RA5	*Injection Volum*	2,00
Injection Type:	Unknown	*Channel:*	UV_VIS_1
Analityk	Dominik Duczmal	*Wavelength:*	230
Instrument Method:	metoda kanna	*Bandwidth:*	4
Processing Method:	Suma wszystkich krzywych	*Dilution Factor:*	100,0000
Injection Date/Time:	27.sie.20 01:10	*Sample Weight:*	131,1000

Chromatogram

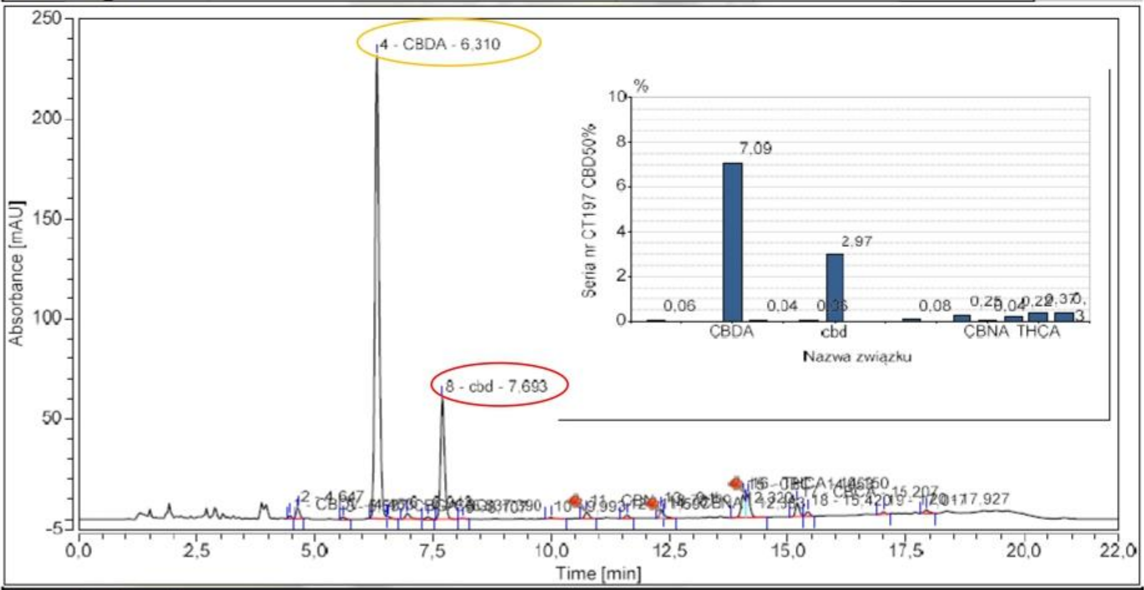

Integration Results

No.	Peak Name	Retention Time<br>min	Area<br>mAU*min	Height<br>mAU	Dilution	Weight<br>mg	Amount<br>ug	Stezenie procentowe<br>%
1	CBDV	4,453	0,107	1,605	100,0	131,1	0,5527	0,06
2		4,647	0,440	5,616	100,0	131,1	n.a.	n.a.
3		5,577	0,090	1,018	100,0	131,1	n.a.	n.a.
4	CBDA	6,310	23,017	231,464	100,0	131,1	70,8969	7,09
5	CBGA	6,537	0,114	1,134	100,0	131,1	0,3634	0,04
6		6,943	0,324	2,786	100,0	131,1	n.a.	n.a.
7	CBG	7,390	0,113	1,136	100,0	131,1	0,6133	0,06
8	cbd	7,693	5,762	60,787	100,0	131,1	29,6787	2,97
9		8,107	0,040	0,425	100,0	131,1	n.a.	n.a.
10		9,993	0,080	0,656	100,0	131,1	n.a.	n.a.
11	CBN	10,737	0,319	3,339	100,0	131,1	0,7794	0,08
12		11,597	0,156	1,667	100,0	131,1	n.a.	n.a.
13	9-thc	12,320	0,403	4,623	100,0	131,1	2,4681	0,25
14	CBNA	12,383	0,103	1,687	100,0	131,1	0,4490	0,04
15	CBC	14,053	0,959	11,084	100,0	131,1	2,2272	0,22
16	THCA	14,150	1,122	11,244	100,0	131,1	3,6608	0,37
17	CBCA	15,207	0,611	6,822	100,0	131,1	3,7653	0,38
18		15,420	0,222	2,398	100,0	131,1	n.a.	n.a.
19		17,017	0,073	1,203	100,0	131,1	n.a.	n.a.

Figure S2. Sample 2 and chromatographic analysis of a cannabinoid extract containing a mixture of cannabinoids.
